# Delta-9-tetrahydrocannabinol increases striatal glutamate levels in healthy individuals: implications for psychosis

**DOI:** 10.1038/s41380-019-0374-8

**Published:** 2019-02-15

**Authors:** Marco Colizzi, Nathalie Weltens, Philip McGuire, David Lythgoe, Steve Williams, Lukas Van Oudenhove, Sagnik Bhattacharyya

**Affiliations:** 1 National Institute for Health Research (NIHR) Biomedical Research Centre (BRC), South London and Maudsley NHS Foundation Trust, and Department of Psychosis Studies, Institute of Psychiatry, Psychology and Neuroscience, King’s College London, London, SE5 8AF UK; 2 Laboratory for Brain-Gut Axis Studies (LaBGAS), Translational Research Center for Gastrointestinal Disorders (TARGID), Department of Chronic Diseases, Metabolism and Ageing, University of Leuven, Leuven, 3000 Belgium; 3 Department of Neuroimaging, Institute of Psychiatry, Psychology and Neuroscience, King’s College London, London, SE5 8AF UK

**Keywords:** Neuroscience, Schizophrenia

## Abstract

The neurobiological mechanisms underlying the association between cannabis use and acute or long-lasting psychosis are not completely understood. While some evidence suggests altered striatal dopamine may underlie the association, direct evidence that cannabis use affects either acute or chronic striatal dopamine is inconclusive. In contrast, pre-clinical research suggests that cannabis may affect dopamine via modulation of glutamate signaling. A double-blind, randomized, placebo-controlled, crossover design was used to investigate whether altered striatal glutamate, as measured using proton magnetic resonance spectroscopy, underlies the acute psychotomimetic effects of intravenously administered delta-9-tetrahydrocannabinol (Δ9-THC; 1.19 mg/2 ml), the key psychoactive ingredient in cannabis, in a set of 16 healthy participants (7 males) with modest previous cannabis exposure. Compared to placebo, acute administration of Δ9-THC significantly increased Glutamate (Glu) + Glutamine (Gln) metabolites (Glx) in the left caudate head (*P* = 0.027). Furthermore, compared to individuals who were not sensitive to the psychotomimetic effects of Δ9-THC, individuals who developed transient psychotic-like symptoms (~70% of the sample) had significantly lower baseline Glx (placebo; *P* 7= 0.023) and a 2.27-times higher increase following Δ9-THC administration. Lower baseline Glx values (*r* = −0.55; *P* = 0.026) and higher previous cannabis exposure (*r* = 0.52; *P* = 0.040) were associated with a higher Δ9-THC-induced Glx increase. These results suggest that an increase in striatal glutamate levels may underlie acute cannabis-induced psychosis while lower baseline levels may be a marker of greater sensitivity to its acute psychotomimetic effects and may have important public health implications.

## Introduction

Cannabis is the most widely used illicit drug in Europe and over the world, with approximately 200 million users [1] and an estimated 13 million individuals with cannabis dependence [2]. It represents a public health concern as cannabis use can induce transient psychotic symptoms [3–5] and trigger the onset of psychosis in vulnerable individuals [6]. Moreover, cannabis use can exacerbate psychotic symptoms [7–9] and increase the risk of relapse [10–12] in patients with established psychosis in a dose-dependent manner [13].

Cannabis exerts its psychotomimetic effects primarily through its psychoactive component delta-9-tetrahydrocannabinol (∆9-THC) [14–16]. ∆9-THC is a partial agonist at the endocannabinoid receptor type 1 (CB1), which is widely expressed throughout the brain [17] and downregulated in response to sustained cannabis use [18]. ∆9-THC has consistently been shown to stimulate the neuronal firing of mesolimbic dopamine neurons and elevate striatal dopamine levels in animal models [19]. However, acute administration of ∆9-THC has been shown to induce striatal dopamine release in some [20–22] but not all human studies [23, 24] (also reviewed in [25]), while a deficit in striatal dopamine release has been reported in cannabis dependence [26]. Additional evidence suggests that ∆9-THC disrupts striatal function [27], and genetic variation in dopamine signaling modulates this effect [16].

The difficulty in capturing the acute effect of Δ9-THC on striatal dopamine in man may be explained by the biological distance between Δ9-THC effects and dopamine dysregulation, as evidence suggests that Δ9-THC does not affect dopamine release directly but via CB1-dependent modulation of glutamate signaling [17]. Converging evidence from preclinical studies indicates that acute Δ9-THC administration induces a dose-dependent increase in cortical extracellular, striatal, and hippocampal intracellular glutamate levels through the activation of CB1 receptors at glutamatergic presynapses in cortical and subcortical brain regions, reflecting a reduction in synaptic glutamate levels and receptor functioning [28–30], also reviewed here [31]. A limited number of studies consistently support the evidence for altered brain glutamate levels as measured by proton magnetic resonance spectroscopy (1H-MRS) in otherwise healthy chronic cannabis users, with all [32–35] but one [36] of the five studies indicating reduced levels of glutamate-derived metabolites Glutamate (Glu) or Glutamate + Glutamine (Glx) in both cortical and subcortical brain areas. The only study not showing an effect of cannabis on glutamate in man investigated a modestly sized sample of cannabis users with concurrent methamphetamine use [36]. In contrast, another study conducted in a larger sample suggested reduced Glx metabolite concentration also in individuals with a history of other illicit drug use [37]. However, the cross-sectional case-control design of these studies does not allow one to infer a cause–effect relationship underlying the observed association between cannabis use and glutamatergic alterations in the brain.

To our knowledge, no study has as yet investigated the acute effect of Δ9-THC on brain glutamate levels in man as a potential mechanism underlying its psychotomimetic effects. Therefore, we employed a placebo-controlled acute pharmacological challenge design to investigate the acute effect of Δ9-THC administration on brain glutamate levels in man. We focused on three brain regions, the striatum, the hippocampus, and the anterior cingulate cortex (ACC), as preclinical studies suggested that acute Δ9-THC administration increased glutamate levels not only in the striatum but also in other brain regions, such as the prefrontal cortex and hippocampus [28–30].

Evidence suggests that (1) Δ9-THC administration in animal models increases glutamate levels in the striatum [30], (2) Δ9-THC-induced increase in glutamate levels leads to an excess striatal dopamine via neuronal circuitry involving hypofunctioning N-methyl-D-aspartate (NMDA) receptors [28], and (3) Δ9-THC-induced modulation of striatal activation is related to the severity of acute psychotomimetic effects induced by it in humans [15, 16, 27]. Hence, we specifically hypothesized that (1) acute ∆9-THC administration would be associated with an increase in striatal glutamate-derived metabolites; (2) ∆9-THC-induced striatal glutamate increase would be associated with the development of psychotomimetic symptoms. Based on the limited evidence of a blunted effect of acute Δ9-THC administration on neurochemical markers (brain-derived neurotrophic factor (BDNF)) in cannabis users compared to healthy subjects [38], the following hypothesis was also tested: (3) previous cannabis exposure would modulate the acute effect of ∆9-THC on striatal glutamate. We also carried out exploratory analyses to examine whether the acute effects of Δ9-THC on brain glutamate levels in man were specific to the striatum or also noted in the hippocampus and ACC.

## Methods

A detailed description of the experimental procedure, psychopathological assessment, image acquisition, 1H-MRS quantification, and statistical analyses is provided in Supplementary Methods and is summarized here briefly.

We employed a double-blind, randomized, placebo-controlled, crossover design, with counterbalanced order of drug administration, using an established protocol [16, 39]. Sixteen right-handed healthy participants (7 males), abstinent from cannabis for at least 6 months and with no history of alcohol abuse, nicotine dependence, or illicit drug use, were assessed on two different occasions separated by at least a 2-week interval, with each session preceded by intravenous administration of Δ9-THC (1.19 mg/2 ml) or saline. A power analysis indicated that a total sample of 16 people would allow detection of a medium effect (*d* = 0.65) with 80% power using a one-tailed paired *t*-test.

Immediately before and at 20 min and 2.5 h after drug administration, psychopathological ratings [40–43] were recorded by an expert clinical researcher.

**Fig. 1:**
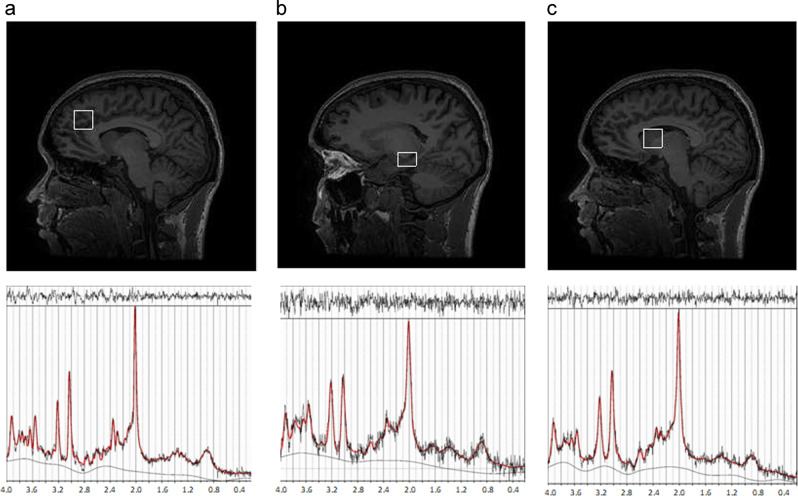
1H-MRS data acquisition. 1H-MRS, proton magnetic resonance spectroscopy. **a** Left anterior cingulate cortex (ACC), **b** left hippocampus, and **c** left head of the caudate

1H-MRS spectra (Point RESolved Spectroscopy—PRESS; TE = 30 ms; TR = 3000 ms; 96 averages) were acquired on a 3 Tesla MR system in the left caudate head, ACC, and hippocampus (Fig. 1), employing the standard GE probe (proton brain examination) sequence, which uses a standardized chemically selective suppression (CHESS) water suppression routine [44] that has been employed before at this centre [45–47]. Data were analyzed with LCModel version 6.3-1L [48].

Data was normally distributed. Paired *t*-tests were used to estimate the effects of ∆9-THC on symptoms (focusing on peak changes from the baseline, as we did not expect symptom manifestation by the 2.5-h time point [14]) and striatal Glx values, and *t*-tests to compare ∆9-THC-induced Glx changes between subjects sensitive to and those not sensitive to the psychotomimetic effects of ∆9-THC, based on the manifestation of clearly detectable primary symptoms of psychosis (≥2-point increase in Positive and Negative Syndrome Scale (PANSS) [40] delusions, hallucinations, unusual thought content, suspiciousness, and grandiosity items), as drawn from previous factor analytic work [49] as well as previous work to characterize acute sensitivity to ∆9-THC [50]. Pearson correlation analyses were used to test for an association between changes in striatal Glx values and previous cannabis exposure (SPSS version 22; SPSS Inc., Chicago, IL).

The composite Glx peak has been widely used as a marker of glutamatergic function, because it likely predominantly reflects glutamate levels, which are typically 5–6 times higher than those of glutamine [51]. Many of the functions of glutamine are connected to the formation of glutamate and the glutamate/glutamine cycle has to be seen as a bi-directional cycle involved in key aspects of metabolism and synaptic function [52]. Research evidence suggests a close coupling of overall neuronal activity and glutamate–glutamine fluxes, with cortical synaptic glutamate release and glutamate–glutamine cycling consuming approximately 60–80% of the energy produced by oxidative metabolism of glucose. This evidence suggests that synaptic glutamate–glutamine cycling cannot be differentiated from overall glutamate metabolism [53]. Therefore, Glx, the main outcome measure of the MRS study presented here, reflects the total glutamatergic pool available for synaptic/metabolic activity [54].

## Results

### Demographic variables, physiological measures, and whole-blood Δ9-THC levels

Study participants had a mean age of 24.44 years (SD: 4.29). They had a mean of 16.94 (SD: 2.84) years of education.

Placebo administration had no effect on systolic (mmHg, *M* ± SD; baseline: 117.31 ± 13.26; drug: 118.56 ± 9.75; *P* > 0.1) and diastolic blood pressure (baseline: 62.5 ± 9.32; drug: 66.38 ± 10.56; *P* > 0.05), and heart rate (beats per minute; baseline: 69.44 ± 13.28; drug: 70.94 ± 13.90, *P* > 0.1). ∆9-THC administration had no effect on systolic (baseline: 117.13 ± 10.35; drug: 117.81 ± 11.78; *P* > 0.1) and diastolic blood pressure (baseline: 64.25 ± 9.28; drug: 66.13 ± 7.05; *P* > 0.1) but a significant effect on heart rate (baseline: 68.69 ± 12.72; drug: 89.31 ± 22.57, *t* = 4.65, *P* < 0.001).

The Δ9-THC plasma levels (*M* ± SE; gas chromatography–mass spectrometry, GC–MS) reached a peak 20 min after drug administration (220.2 ± 34.1 ng/mL), and then began to fall (2.5 h after drug administration: 54.6 ± 8.6 ng/mL).

### Acute effect of Δ9-THC on psychopathological measures

**Fig. 2:**
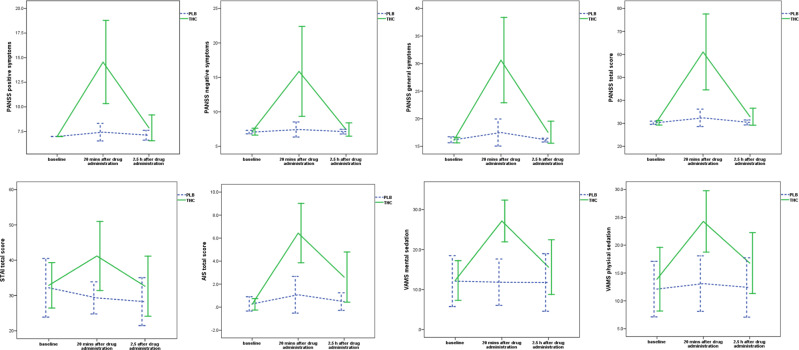
Acute effect of Δ9-THC on psychopathological measures. THC (−)-trans-Δ^9^-tetrahydrocannabinol, PLB placebo, PANSS Positive and Negative Syndrome Scale, STAI State-Trait Anxiety Inventory, AIS Analog Intoxication Scale, VAMS Visual Analog Mood Scale, mins minutes, h hours, *P* 2-tailed; error bars show standard deviations

As expected, administration of Δ9-THC was associated with acute induction of transient psychotic symptoms (PANSS positive symptoms subscale, *t* = 6.62, *P* < 0.001; PANSS negative symptoms subscale, *t* = 4.95, *P* < 0.001; PANSS general symptoms subscale, *t* = 6.85, *P* < 0.001; PANSS total score, *t* = 6.77, *P* < 0.001). Also, Δ9-THC induced an acute and transient increase in symptoms of anxiety (STAI scale, *t* = 3.72, *P* = 0.002). Finally, subjects experienced significant Δ9-THC-induced intoxication (AIS, *t* = 9.41, *P* < 0.001) and sedation (VAMS mental sedation subscale, *t* = 7.72, *P* < 0.001; VAMS physical sedation subscale, *t* = 4.90, *P* < 0.001; Fig. 2). Eleven subjects (69%) were identified as sensitive to the psychotomimetic effects of ∆9-THC as determined on the basis of ≥2-point increase in the relevant PANSS items (as described in Methods) [49]. They had a 5.91 (±4.18) point increase in the primary symptoms of psychosis compared to a 0.6 point increase (±0.55) for the remaining subjects (drug effect, *t* = 4.13, *P* = 0.002).

### 1H-MRS results

Voxel segmentation and spectral quality are reported in Table 1.

**Table 1:** Voxel segmentation and spectral quality

Brain region	Δ9-THC	PLB	Statistics	
Parameter	*M* (SD)	*M* (SD)	*t*	*P* value
**Left caudate head**				
Cramér–Rao lower bound				
* Glu*	9.25 (2.35)	9.00 (1.83)	0.37	0.71
* Glx*	11.12 (3.54)	10.87 (2.58)	0.24	0.81
* NAA* + *NAAG*	3.62 (1.15)	3.19 (0.54)	1.52	0.15
* Cr*	3.37 (0.62)	3.50 (0.89)	−0.56	0.58
* mI*	8.07 (3.47)	9.67 (4.67)	−1.50	0.15
* GPC* + *PCh*	4.31 (0.79)	4.44 (1.46)	−0.34	0.74
Full width at half maximum	0.07 (0.01)	0.06 (0.01)	1.91	0.08
Signal to noise ratio	16.50 (3.92)	17.88 (3.69)	−1.14	0.27
Grey matter (%)	48.77 (7.05)	49.32 (5.65)	−0.28	0.78
White matter (%)	49.40 (7.26)	48.84 (6.73)	0.31	0.76
Cerebrospinal fluid (%)	1.81 (1.86)	1.80 (1.79)	0.02	0.99
**Left anterior cingulate cortex**				
Cramér–Rao lower bound				
* Glu*	5.69 (0.79)	5.94 (1.06)	−0.77	0.45
* Glx*	6.31 (0.87)	6.75 (1.06)	−1.81	0.09
* NAA* + *NAAG*	2.69 (0.48)	2.69 (0.60)	0.00	1.00
* Cr*	2.75 (0.45)	2.62 (0.62)	0.70	0.50
* mI*	4.69 (0.60)	5.12 (1.89)	−0.92	0.37
* GPC* + *PCh*	3.25 (0.58)	3.25 (0.45)	0.00	1.00
Full width at half maximum	0.04 (0.01)	0.03 (0.01)	1.78	0.10
Signal to noise ratio	24.56 (5.20)	25.44 (5.50)	−0.61	0.55
Grey matter (%)	67.64 (4.43)	67.06 (5.14)	0.94	0.36
White matter (%)	10.94 (2.59)	11.85 (2.46)	−1.59	0.13
Cerebrospinal fluid (%)	21.26 (5.36)	20.94 (5.36)	0.45	0.66
**Left hippocampus**				
Cramér–Rao lower bound				
* Glu*	9.87 (1.82)	9.94 (2.52)	−0.10	0.92
* Glx*	9.50 (2.85)	10.31 (2.73)	−0.90	0.38
* NAA* + *NAAG*	4.00 (1.21)	4.75 (1.13)	−2.16	0.05
* Cr*	4.06 (0.68)	4.19 (0.75)	−0.81	0.43
* mI*	5.37 (1.09)	6.06 (1.65)	−2.11	0.05
* GPC* + *PCh*	4.06 (0.44)	4.19 (0.83)	-0.62	0.54
Full width at half maximum	0.07 (0.01)	0.07 (0.01)	0.95	0.36
Signal to noise ratio	12.81 (2.14)	12.13 (2.28)	1.14	0.27
Grey matter (%)	59.86 (8.12)	60.23 (6.58)	−0.20	0.85
White matter (%)	36.42 (8.96)	35.68 (7.60)	0.36	0.72
Cerebrospinal fluid (%)	3.69 (1.39)	4.06 (1.56)	−1.39	0.19

*Δ9-THC* delta-9-tetrahydrocannabinol, *PLB* placebo, *Glu* glutamate, *Glx* glutamate + glutamine, *NAA* *+* *NAAG* N-acetylaspartate + N-acetylaspartylglutamate, *Cr* creatine, *mI* myo-inositol, *GPC* *+* *PCh* Glycerophosphocholine + phosphocholine

### Striatal Glx measures

**Fig. 3:**
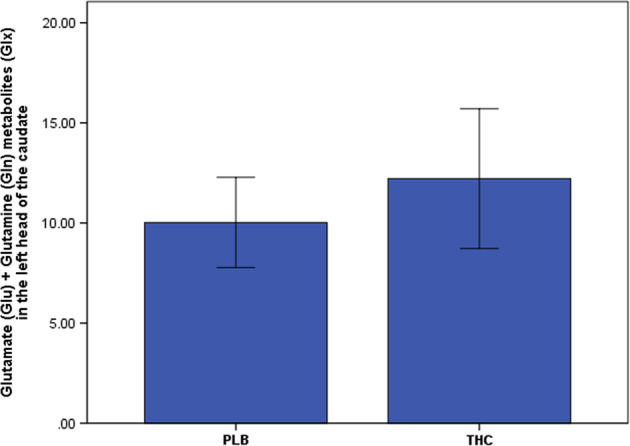
Acute effect of Δ9-THC on glutamate measures in the left head of the caudate. THC (−)-trans-Δ^9^-tetrahydrocannabinol, PLB placebo, *P* 1-tailed; error bars show standard deviations

As hypothesized, acute ∆9-THC administration increased Glutamate (Glu) + Glutamine (Gln) metabolites (Glx) in the left caudate head (placebo: 10.03 ± 2.25; ∆9-THC: 12.22 ± 3.49; *t* = 2.09, *P* = 0.027; effect size: 0.75; Fig. 3). There was an inverse relationship between baseline Glx values (as measured under the placebo condition) and change in Glx induced by acute ∆9-THC administration. This was such that, the lower the Glx values under placebo, the higher was the increase following ∆9-THC administration (*r* = −0.55; *P* = 0.026). Furthermore, there was a positive correlation between previous cannabis exposure (as indexed using lifetime number of times of cannabis use) and ∆9-THC-induced increase in Glx (drug effect, *r* = 0.52; *P* = 0.040).

**Fig. 4:**
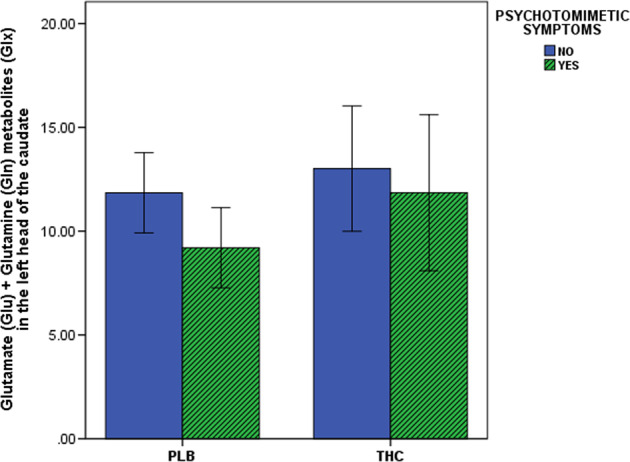
Acute effect of Δ9-THC on glutamate measures in the left head of the caudate as a function of the psychotomimetic symptom manifestation. THC (−)-trans-Δ^9^-tetrahydrocannabinol, PLB placebo, *P* 2-tailed; error bars show standard deviations

Glx values under the placebo condition were significantly lower in subjects who were sensitive to ∆9-THC-induced psychotomimetic effects (9.20 ± 1.93) compared to subjects who were not (11.85 ± 1.93; *t* = 2.54, *P* = 0.023). Following acute ∆9-THC administration, compared to subjects who were not sensitive to the psychotomimetic effects (13.01 ± 3.02), subjects sensitive to the psychotomimetic effects of ∆9-THC had a 2.27-times higher increase in Glx values (11.85 ± 3.76). However, this difference failed to reach significance (*P* > 0.1; Fig. 4).

Acute ∆9-THC administration had no effect on ACC (placebo: 19.47 ± 3.04; ∆9-THC: 20.11 ± 2.04) and hippocampal Glx (placebo: 11.74 ± 2.32; ∆9-THC: 12.29 ± 2.30; all *P* > 0.1). Other metabolite levels are reported in Supplementary Table 1.

## Discussion

This is the first human study to investigate the acute effect of intravenous Δ9-THC administration on brain glutamate levels, and whether glutamate level alterations underlie the acute psychotomimetic effects of Δ9-THC. Consistent with our first hypothesis and with previous evidence from animal studies [28–31] we found that acute administration of Δ9-THC significantly increased Glx levels in the left caudate head compared to the placebo. As predicted, we also found that this was associated with transient psychotomimetic effects induced by Δ9-THC. Our most novel finding is that individuals who experienced transient psychotomimetic effects following Δ9-THC (~70% of the sample) had significantly lower baseline Glx (as under placebo) and an almost two-and-a-half-fold higher increase in Glx following Δ9-THC administration compared to individuals who were not sensitive to the psychotomimetic effects of Δ9-THC. Finally, consistent with our prediction, previous cannabis exposure was positively associated with Δ9-THC-induced Glx increase. Exploratory analyses also suggested that the acute effects of Δ9-THC on human brain Glx levels are region-specific, as Δ9-THC administration increased Glx levels in the striatum, but not in the hippocampus or the ACC.

In addition to the brain stem projections, the key inputs to the striatum are mesolimbic dopaminergic (DA) projections from the ventral tegmental area (VTA) as well as cortical and thalamic glutamatergic projections [55, 56]. The activity of VTA DA neurons in vivo is dominated by pacemaker-like tonic firing interrupted by phasic bursts leading to striatal dopamine release [57], and this shift from tonic pacemaker firing to bursting is strongly controlled by synaptic input from glutamatergic and GABAergic afferents to the dopamine neurons [58]. Upon acute exposure, endocannabinoids regulate synaptic strength by acting on glutamatergic afferents to VTA dopamine neurons via activation of CB1 receptors [59]. Preclinical evidence also indicates that the intravenous administration of Δ9-THC can increase VTA dopamine neuronal activity, being ultimately responsible for an increase in striatal dopamine levels through the mesolimbic pathway [19]. However, in vitro studies have demonstrated that cannabinoids do not affect dopamine concentrations when locally applied in the striatum [60], while increasing dopaminergic firing when administered in the VTA [61]. Since VTA dopamine neurons do not express CB1 receptor protein nor mRNA [62], this argues against a direct effect of cannabinoids on dopamine neuron activity, also potentially accounting for the inconsistent evidence on effects of Δ9-THC administration on striatal dopamine release in man [20–25]. In contrast, the glutamatergic inputs to the striatum are especially relevant as they are involved in the processing of different stimuli, such as rewarding and stressful information, and the selection of related behavioral responses [63]. In line with this, acute Δ9-THC administration in animal models has been shown to consistently increase glutamate levels in a number of brain regions including the striatum [28–31]. Therefore, glutamate rather than dopamine may play a more important role in the neurochemical underpinnings of the acute psychotomimetic effects of cannabis [31]. However, no acute challenge study had investigated the effect of acute administration of Δ9-THC on glutamate metabolism in humans. In sum, our study confirms preclinical evidence [28–31] that a single dose of Δ9-THC may increase striatal glutamate levels and suggests this as a potential mechanism underlying the acute psychotomimetic effects of cannabis.

Previous preclinical research indicates that a history of psychostimulant self-administration leads to decreased basal glutamate in both the striatum and the primary neuronal source of striatal glutamate, the prefrontal cortex. Instead, acute psychostimulant drug administration in abstinent animal models previously exposed to the drug induces an enhancement of cortical and striatal glutamate release not seen in drug-naive subjects [64, 65]. Drug-induced heightened activation of cortical glutamatergic afferents to the VTA has been proposed to modulate behavioral sensitization and addiction [66]. In line with this evidence, we found that the lower the striatal Glx levels at baseline, the higher was the increase after Δ9-THC administration. Furthermore, we found that the higher the previous cannabis exposure, the higher was the Δ9-THC-induced striatal increase, potentially suggesting sensitization due to the effects of previous cannabis exposure. However, an important caveat to such an interpretation is the relatively modest levels of previous cannabis use (1 ≤ previous use ≤60 times, 10.4 ± 14.4 times on average). Nevertheless, sensitization (if it is present) to such low levels of exposure is not surprising as preclinical evidence suggests that even a single exposure to psychostimulant drugs can be sufficient to induce long-lasting behavioral and cellular sensitization [66]. One may therefore speculate that the glutamatergic system may have a different steady-state homeostasis as a function of previous exposure, from where the system is particularly susceptible to destabilizing influences that may affect it, such as the acute administration of Δ9-THC. As lower baseline Glx levels and related higher Δ9-THC-induced increase were evident in individuals who developed psychotomimetic symptoms under Δ9-THC compared to individuals who did not, our findings suggest that Δ9-THC-induced psychotomimetic symptoms could be explained by Δ9-THC effects on tonic (basal) versus phasic (burst) glutamate system function, which in turn are modulated by previous cannabis use.

ACC and hippocampal Glx were not significantly increased by acute Δ9-THC administration, suggesting that glutamate alteration in the striatum may represent a specific locus of abnormality underlying sensitivity to the acute and transient psychotomimetic effects of Δ9-THC. However, this needs to be confirmed in larger samples.

Glutamate steady-state homeostasis and sensitization may also account for the apparent discrepancy between the reduction in glutamate observed in studies of chronic cannabis use in man [32–35, 37] and the increase in glutamate observed in preclinical acute challenge studies [28–31] as well as in the present acute challenge study in humans. Animal studies of chronic psychostimulant use clearly indicate drug-induced changes in glutamate regulation, such that basal glutamate levels are decreased while glutamate release is enhanced during drug exposure [64, 65]. Cannabis use may involve progressive neurochemical adaptations in glutamate function, which need to be further investigated. Determining the regional changes in glutamate function that may result from repeated cannabis exposure is also imperative to understanding their relevance to the acute and chronic psychoactive effects of cannabis use. To date, there is robust evidence for altered glutamate steady-state homeostasis in animal models of addiction [64, 65]. However, aberrant glutamate function has also been suggested in psychosis and related disorders. In particular, a systematic review of 63 studies investigating metabolite biomarkers of schizophrenia has indicated glutamate increase as one of the most consistent potential metabolite signatures of the disorder [67]. Increased striatal glutamate levels have also been described in subjects at ultra-high risk for psychosis, and they are also associated with conversion to psychosis [68, 69].

The major strength of this study is its design. Study subjects were recruited if they had a minimal history of cannabis use, had been abstinent from cannabis for at least 6 months, and had negligible use of other substances (alcohol, tobacco, and other illicit drugs). Therefore, we can reasonably rule out the possibility that some of the results observed could be attributed to the effects of other substance use or cannabis withdrawal, dependence, or intoxication. Moreover, for each study participant, there was an interval of at least 14 days between the two study visits. This helps exclude the possibility of any carryover effects as Δ9-THC has been shown to have an elimination half-life of 18 h to 4.3 days [70]. Also, all the participants’ urine samples collected at each study visit at baseline were negative for the presence of Δ9-THC. However, these strict inclusion criteria, while offering advantages in terms of a controlled sample, may at the same time limit the generalizability of the present results to the wider population of cannabis users. Also, the intravenous route for Δ9-THC administration allowed much more consistent Δ9-THC blood levels across study subjects [14], but might have similarly affected the generalizability of the results to the effects of recreational cannabis use. Another limitation of the present study is that, due to its design, it was not possible to examine the test–retest reliability of the MRS Glx measure for the regions investigated. However, evidence indicates that GM Glx in healthy subjects has relatively high reproducibility and test–retest reliability at 3 Tesla [71]. Furthermore, the within-subject design helped avoid the confounding effect of between-subject differences in the outcome variable [71].

It is worth noting that 1H-MRS does not allow us to disentangle whether measured glutamate is from the neurotransmitter or the metabolic pool. Nevertheless, research evidence indicates that majority of the brain glutamate is cycled through the neurotransmitter pool [53] and 1H-MRS-related glutamate measures are likely to be related to glutamatergic neurotransmission [54].

It is worth considering a few other potential alternative explanations for the results presented here, such as effect of spectral quality differences, effect of T2 relaxation and test–retest effect. Cramér–Rao lower-bound values were considerably below the 20% threshold under both drug conditions. Although full width at half maximum (FWHM) values showed a trend toward difference between the two drug conditions, they were also within the spectral quality recommended by Kreis (FWHM of metabolites < 0.07–0.1 ppm) [72]. Collectively, they suggest good quality data, and are more informative than a comparison of quality measures across the drug conditions (∆9-THC, placebo) [72]. Results presented here also point toward regional specificity of the acute effects of THC, as no effects on any metabolite were observed in the hippocampus and anterior cingulate, arguing against these effects being a result of spectral quality differences.

With reference to the possibility that a T2 relaxation effect might have occurred, decreasing the signal, this is expected to happen when a longer echo time (TE) is used [73]. Instead, the combination of a short TE and a long repetition time (TR) used in this study is considered to allow the acquisition of signals with minimal signal loss due to T2- and T1-weighting [73]. Nevertheless, the T2 relaxation of water, which differs between white matter, grey matter, and CSF may be different between individuals because of the presence of different fractions of these components in the MRS voxel, and arguably may introduce a systematic bias in metabolite quantification due to group difference in T2 relaxation. However, this is unlikely to have systematically affected the results of the present study as we employed a within-subject repeated measures design, thereby mitigating the effect of group difference in the fraction of grey matter, white matter, and CSF in the MRS voxel contributing to difference in T2 relaxation under the two drug conditions (∆9-THC versus Placebo). Furthermore, the randomized crossover design with counterbalanced order of drug administration employed in this study also helped mitigate a possible test–retest effect.

In summary, this study suggests that striatal glutamate levels are increased following a single dose of ∆9-THC in healthy individuals. This ∆9-THC-induced glutamate increase likely underlies the acute cannabis-induced psychotomimetic effects, as it seems to be specific to subjects experiencing psychotomimetic effects. These results also suggest that lower baseline levels of striatal glutamate may be a marker of sensitivity to the acute psychotomimetic effects of cannabis and potential sensitization to the modulation of striatal glutamate levels by ∆9-THC as a function of previous cannabis exposure may develop early. Collectively, these results provide novel insight into the neurochemical underpinnings of the effects of cannabis in man and may point towards potential approaches towards mitigating the adverse effects of cannabis, which may have important public health implications.

## Supplementary information

Supplementary Methods

Supplementary Table 1
