# Supplementary material for: Delta-9-tetrahydrocannabinol increases striatal glutamate levels in healthy individuals: implications for psychosis
Source: Mol Psychiatry. 2019 Feb 15;25(12):3231–40. doi: 10.1038/s41380-019-0374-8 (PMC7714685; doi:10.1038/s41380-019-0374-8)
Supplement: Supplementary file 1 — Supplementary Methods [file 41380_2019_374_MOESM1_ESM.doc]

**SUPPLEMENTARY METHODS**

**Experimental procedure**

Prior to each study visit, participants were advised to get at least 6 to 8 hours sleep overnight and to refrain from smoking for 4 hours, to take caffeine for 12 hours and alcohol for 24 hours. Also, subjects had been abstinent from cannabis for at least 6 months before the first study visit and were advised to abstain from using any substance throughout the duration of the study. On arrival at the study centre in the morning, participants had a light standardized breakfast after an overnight fast. All subjects had a negative urinary drug screen for amphetamines, benzodiazepines, cocaine, opiates, and delta-9-tetrahydrocannabinol (Δ9-THC), tested on each study day using immunometric assay kits. All female participants had a negative pregnancy test; also, all of them were consistently using a reliable contraceptive method, apart from a single subject who underwent both study visits in the first week of the menstrual cycle. After physical examination, an indwelling intravenous line was placed in the nondominant arm. This cannula was used for the intravenous administration of Δ9-THC (1.19 mg/ 2 ml, ≥ 99% pure; THC-Pharm, Frankfurt, Germany, [http://biochem.thc-pharm.de](http://biochem.thc-pharm.de/); pharmaceutical formulation at the Barts Health NHS Trust pharmacy according to previous work (1)) or saline as well as blood collection at different time points before and after drug challenge. A dose of 1.19 mg was used, as previous work has suggested that an intravenous dose range 0.015-0.03 mg/ kg body weight is consistently associated with the induction of psychotomimetic effects (2). Heart rate and blood pressure were monitored via a digital recorder and an automated arm cuff for the entire duration of the study.

**Subjects**

Sixteen healthy, English-speaking, right-handed individuals participated in this study. Demographic information such as age, ethnicity, and level of education was recorded. All except 3 (mixed ethnicity) of the volunteers were white Europeans. All subjects gave written, informed consent, and completed all of the components of the study. Personal or family history of psychiatric illness in first-degree relatives represented an exclusion criterion. None of the subjects included in the study had used more than 21 units/week of alcohol on a regular basis. Only 3 subjects had a regular smoking habit (2 of them smoking < 10 cigarettes/ day and 1 smoking 2 cigarettes/ week), 6 had smoked occasionally/ experimentally, and 7 had never smoked. Apart from 3 subjects who had a single experimental use of MDMA, all the remaining participants had never used any other illicit substance. Regarding previous lifetime cannabis exposure, 9 subjects had used cannabis ≤ 5 times, 3 subjects ≤ 10 times, 2 subjects ≤ 20 times, 1 subject 20 times, and 1 subject 60 times; 10.4 ± 14.4 times on average).

**Psychopathological assessment**

All participants were interviewed at baseline by a psychiatrist using the Structured Clinical Interview for DSM-5 (SCID-5) as a guide for the assessment of the psychotic spectrum disorders (3). On each study day, the following assessments were carried out immediately before and at 20 mins and 2.5 hours after drug administration. First, the Positive and Negative Syndrome Scale (4) (PANSS) and the State-Trait Anxiety Inventory (STAI) (5) were used in order to assess change in psychotic and anxiety symptoms. Second, sedation and intoxication were assessed using the Visual Analog Mood Scale (6) (VAMS), and Analog Intoxication Scale (7) (AIS) respectively. Finally, participants were contacted the day after each study visit for a health check as part of the study standard operating procedure (SOP). Symptoms lasting longer than expected or occurring after the end of the study visit were also recorded.

**Image acquisition**

All subjects underwent structural MRI, functional MRI (fMRI) and proton magnetic resonance spectroscopy (1H-MRS) scanning in both sessions. Images were acquired on a General Electric (Milwaukee, Wisconsin) 3.0 Tesla HDx MR system 30 mins following drug administration in a session lasting a maximum of 120 min. The present report focuses on 1H-MRS data.

***Structural MRI***

Structural images were acquired using a whole-brain three-dimensional sagittal T1-weighted scan, with parameters based on the Alzheimer’s Disease Neuroimaging Initiative (ADNI) (TE = 2.85 ms; TR = 6.98 ms; inversion time = 400 ms; flip angle = 11º; voxel size 1.0x1.0x1.2 mm; for full details see http://adni.loni.usc.edu/methods/mri-analysis/mri-acquisition/).

***1H-MRS***

1H-MRS spectra (PRESS - Point RESolved Spectroscopy; TE = 30 ms; TR = 3000 ms; 96 averages) were acquired in the left caudate head, anterior cingulate cortex (ACC), and hippocampus, as previously described by Stone (8). We employed the standard GE probe (proton brain examination) sequence, which uses a standardised chemically selective suppression (CHESS) water suppression routine. For each metabolite spectrum, unsuppressed water reference spectra (16 averages) were also acquired as part of the standard acquisition. Shimming and water suppression were optimised, with auto-prescan performed twice before each scan. Using standardised protocols, regions of interest (ROIs) (left caudate head, 20x20x20 mm; left ACC, 20x20x20 mm; left hippocampus, 20x20x15 mm; right-left, anterior-posterior, superior-inferior) were prescribed from the structural T1 scan (Figure 1).

**1H-MRS quantification**

All spectra were analysed with LCModel version 6.3-1L (9) using a standard basis set of 16 metabolites (L-alanine, aspartate, creatine, phosphocreatine, GABA, glucose, glutamine, glutamate, glycerophosphocholine, glycine, myo-inositol, L-lactate, N-acetylaspartate, N-acetylaspartylglutamate, phosphocholine, and taurine), acquired with the same field strength (3 Tesla), localisation sequence (PRESS), and echo time (30 ms). Model metabolites and concentrations used in the basis set are fully detailed in the LCModel manual (http://s-provencher-.com/pages/lcmmanual.shtml). Poorly fitted metabolite peaks (Cramer-Rao minimum variance bounds of >20% as reported by LCModel) were excluded from further analysis. Values of the combined water-scaled measure of glutamate and glutamine (Glx, metabolite of interest) as well as other metabolites were corrected for the voxel tissue composition by using the formula: Mcorr = M*[WM + (1.28*GM) + (1.55*CSF)] / (WM+GM), where M is the uncorrected metabolite value, and WM, GM and CSF are the white matter, grey matter and CSF fractions of the ROI, respectively (10). These fractions were determined for each subject from the structural T1 scans, which were used to localise the spectroscopy ROIs and subsequently segmented into grey matter, white matter, and CSF using SPM8.

**Statistical analyses**

Symptomatic effects were estimated by computing change from the baseline for each drug condition (∆9-THC, placebo) and compared using paired *t*-tests. Subjects were also dichotomised as sensitive to the psychotomimetic effects of ∆9-THC based on the manifestation of clearly detectable primary symptoms of psychosis (≥ 2 point increase at PANSS delusions, hallucinations, unusual thought content, suspiciousness, and grandiosity items), as drawn from previous extensive factor analytic work (described in detail here (11)) as well as previous work to characterize acute sensitivity to ∆9-THC (12). Glx values in the left caudate head obtained during the 2 study sessions were compared using paired *t*-tests and drug effect was obtained by subtracting the values under the placebo condition from the Δ9-THC condition. Also, *t*-tests were used to compare ∆9-THC and placebo Glx values in the left caudate head between subjects sensitive and not sensitive to the psychotomimetic effects of ∆9-THC. Correlation analyses of drug effect were used to test for an association between changes in caudate glutamate values and previous cannabis exposure (SPSS version 22; SPSS Inc, Chicago, Illinois). The same statistics were used to conduct the exploratory analyses on Glx values in the hippocampus and anterior cingulate cortex.

**Ethics**

All procedures contributing to this work comply with the ethical standards of the relevant national and institutional committees on human experimentation and with the Helsinki Declaration of 1975, as revised in 2008.

**REFERENCES**

1. Naef M, Russmann S, Petersen-Felix S, Brenneisen R. Development and pharmacokinetic characterization of pulmonal and intravenous delta-9-tetrahydrocannabinol (THC) in humans. Journal of pharmaceutical sciences. 2004;93(5):1176-84.

2. Radhakrishnan R, Wilkinson ST, D'Souza DC. Gone to pot - a review of the association between cannabis and psychosis. Frontiers in Psychiatry. 2014;5.

3. First MB, Williams JBW, Karg RS, Spitzer RL. Structured Clinical Interview for DSM-5 Disorders, Clinician Version (SCID-5-CV). Arlington, VA.: American Psychiatric Association; 2015.

4. Kay SR, Fiszbein A, Opler LA. The positive and negative syndrome scale (PANSS) for schizophrenia. Schizophr Bull. 1987;13(2):261-76.

5. Guillen-Riquelme A, Buela-Casal G. [Meta-analysis of group comparison and meta-analysis of reliability generalization of the State-Trait Anxiety Inventory Questionnaire (STAI)]. Rev Esp Salud Publica. 2014;88(1):101-12.

6. Folstein MF, Luria R. Reliability, validity, and clinical application of the Visual Analogue Mood Scale. Psychological medicine. 1973;3(4):479-86.

7. Mathew RJ, Wilson WH, Humphreys DF, Lowe JV, Wiethe KE. Regional cerebral blood flow after marijuana smoking. J Cereb Blood Flow Metab. 1992;12(5):750-8.

8. Stone JM. Imaging the glutamate system in humans: relevance to drug discovery for schizophrenia. Curr Pharm Des. 2009;15(22):2594-602.

9. Provencher SW. Estimation of metabolite concentrations from localized in vivo proton NMR spectra. Magnetic resonance in medicine. 1993;30(6):672-9.

10. Modinos G, Simsek F, Azis M, Bossong M, Bonoldi I, Samson C, et al. Prefrontal GABA levels, hippocampal resting perfusion and the risk of psychosis. Neuropsychopharmacology : official publication of the American College of Neuropsychopharmacology. 2018.

11. Fulford D, Pearson R, Stuart BK, Fisher M, Mathalon DH, Vinogradov S, et al. Symptom assessment in early psychosis: the use of well-established rating scales in clinical high-risk and recent-onset populations. Psychiatry Res. 2014;220(3):1077-83.

12. Bhattacharyya S, Sainsbury T, Allen P, Nosarti C, Atakan Z, Giampietro V, et al. Increased hippocampal engagement during learning as a marker of sensitivity to psychotomimetic effects of delta-9-THC. Psychological medicine. 2018:1-9.
