# Supplementary material for: Delta-9-tetrahydrocannabinol increases striatal glutamate levels in healthy individuals: implications for psychosis
Source: Mol Psychiatry. 2019 Feb 15;25(12):3231–40. doi: 10.1038/s41380-019-0374-8 (PMC7714685; doi:10.1038/s41380-019-0374-8)
Supplement: Supplementary file 2 — Supplementary Table 1 [file 41380_2019_374_MOESM2_ESM.docx]

Supplementary Table 1. Metabolite levels

| **Brain region** | **Δ9-THC** | **PLB** | **Statistics** | |
| --- | --- | --- | --- | --- |
| Brain Metabolite | M (SD) | M (SD) | t | P value |
| **Left Caudate Head** | | |  |  |
| Glu | 9.39 (1.91)  1.12 (0.30) | 8.53 (1.43)  1.12 (0.28) | 1.37  -0.01 | 0.191  0.990 |
| *Corrected for voxel tissue content* |  |  |  |  |
| *Scaled to creatine* |  |  |  |  |
| Glx  *Corrected for voxel tissue content*  *Scaled to creatine* | 12.22 (3.49)  1.47 (0.55) | 10.03 (2.25)  1.31 (0.35) | 2.09  1.15 | 0.027*  0.268 |
| NAA+NAAG  *Corrected for voxel tissue content*  *Scaled to creatine* | 11.05 (0.93)  1.30 (0.14) | 10.42 (1.38)  1.33 (0.13) | 1.98  -0.68 | 0.067  0.504 |
| Cr  *Corrected for voxel tissue content* | 8.57 (1.13) | 7.84 (0.99) | 2.65 | 0.018 |
| mI  *Corrected for voxel tissue content*  *Scaled to creatine* | 5.10 (2.01)  0.59 (0.21) | 4.27 (1.74)  0.54 (0.21) | 1.69  0.91 | 0.112  0.374 |
| GPC+PCh  *Corrected for voxel tissue content*  *Scaled to creatine* | 2.06 (0.42)  0.24 (0.04) | 1.91 (0.38)  0.24 (0.04) | 1.47  -0.29 | 0.162  0.776 |
| **Left Anterior Cingulate Cortex** | | | | |
| Glu  *Corrected for voxel tissue content*  *Scaled to creatine* | 15.64 (1.35)  1.45 (0.11) | 15.49 (2.47)  1.43 (0.22) | 0.22  0.40 | 0.827  0.696 |
| Glx  *Corrected for voxel tissue content*  *Scaled to creatine* | 20.11 (2.04)  1.87 (0.16) | 19.47 (3.04)  1.80 (0.26) | 0.90  1.19 | 0.382  0.252 |
| NAA+NAAG  *Corrected for voxel tissue content*  *Scaled to creatine* | 15.44 (0.74)  1.44 (0.11) | 15.54 (1.32)  1.43 (0.11) | -0.31  0.17 | 0.758  0.871 |
| Cr  *Corrected for voxel tissue content* | 10.77 (0.74) | 10.84 (0.61) | -0.49 | 0.627 |
| mI  *Corrected for voxel tissue content*  *Scaled to creatine* | 8.32 (0.90)  0.77 (0.08) | 7.99 (1.26)  0.74 (0.10) | 0.82  0.96 | 0.427  0.350 |
| GPC+PCh  *Corrected for voxel tissue content*  *Scaled to creatine* | 2.76 (0.33)  0.26 (0.03) | 2.74 (0.39)  0.25 (0.04) | 0.26  0.34 | 0.801  0.740 |
| **Left Hippocampus** | | |  | |
| Glu  *Corrected for voxel tissue content*  *Scaled to creatine* | 8.21 (1.45)  1.09 (0.24) | 8.46 (1.71)  1.13 (0.21) | -0.47  -0.63 | 0.646  0.539 |
| Glx  *Corrected for voxel tissue content*  *Scaled to creatine* | 12.29 (2.30)  1.62 (0.37) | 11.74 (2.32)  1.56 (0.24) | 0.75  0.67 | 0.465  0.510 |
| NAA+NAAG  *Corrected for voxel tissue content*  *Scaled to creatine* | 10.25 (1.29)  1.34 (0.13) | 9.82 (1.72)  1.30 (0.14) | 1.41  1.26 | 0.177  0.229 |
| Cr  *Corrected for voxel tissue content* | 7.66 (0.83) | 7.54 (1.05) | 0.50 | 0.624 |
| mI  *Corrected for voxel tissue content*  *Scaled to creatine* | 7.06 (1.43)  0.92 (0.12) | 6.67 (0.99)  0.89 (0.10) | 1.06  0.86 | 0.304  0.402 |
| GPC+PCh  *Corrected for voxel tissue content*  *Scaled to creatine* | 2.65 (0.47)  0.35 (0.05) | 2.53 (0.45)  0.34 (0.04) | 1.06  1.06 | 0.307  0.305 |

Δ9-THC, delta-9-tetrahydrocannabinol; PLB, placebo; CRLB, Cramér–Rao lower bound; Glu, Glutamate; Glx, Glutamate + Glutamine; NAA+NAAG, N-acetylaspartate + N-acetylaspartylglutamate; Cr, Creatine; mI, myo-inositol; GPC+PCh, Glycerophosphocholine + Phosphocholine; * 1-tailed
